# Supplementary figures and images for: Extraneural infection route restricts prion conformational variability and attenuates the impact of quaternary structure on infectivity
Source: PLoS Pathog. 2024 Jul 8;20(7):e1012370. doi: 10.1371/journal.ppat.1012370 (PMC11257401; doi:10.1371/journal.ppat.1012370)

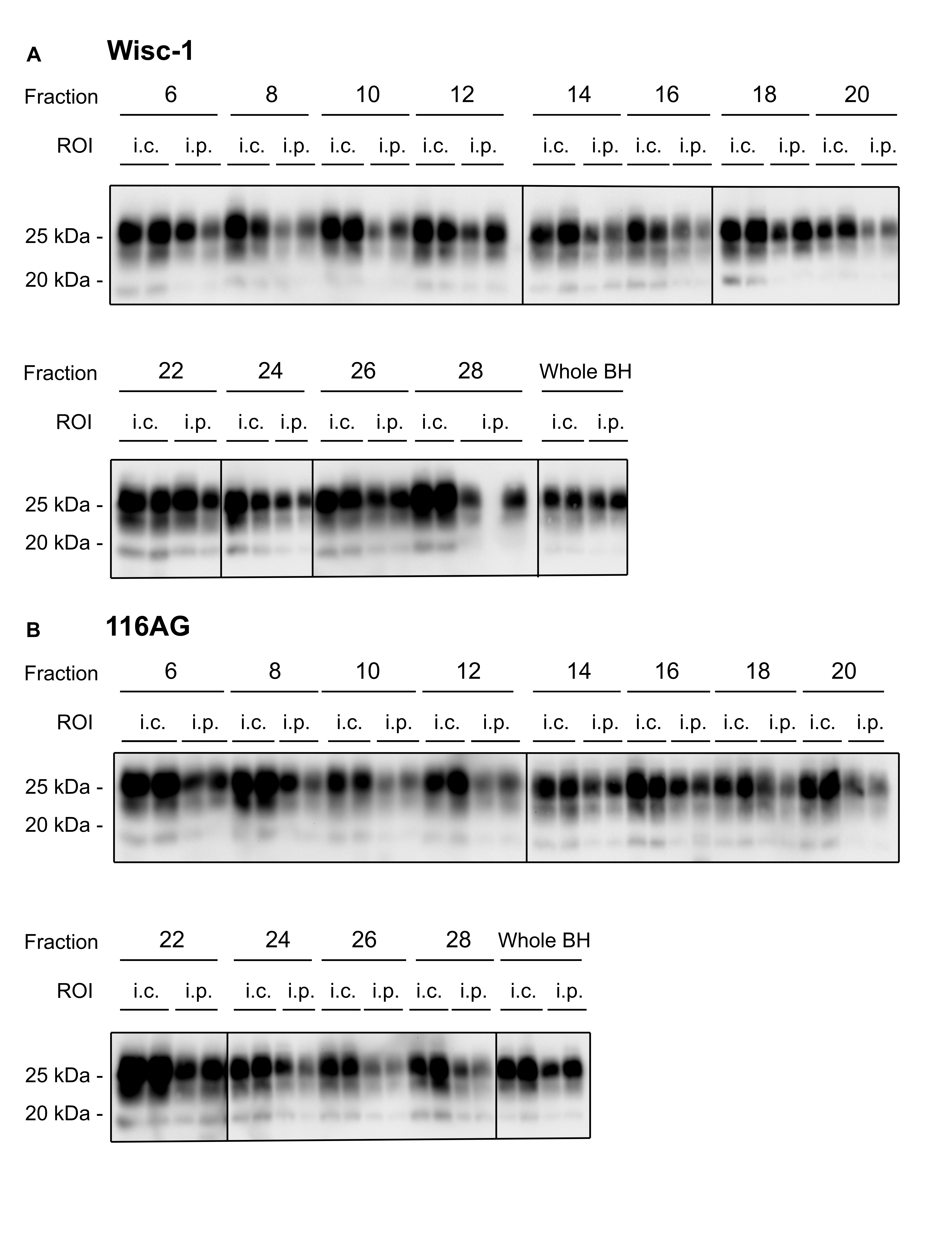

Supplement: S5 Fig — Representative blots of PrPSc from mice inoculated i.c. or i.p. with (A) Wisc-1 or (B) 116AG fractions digested with 50 μg/ml of PK. (TIFF) [file ppat.1012370.s005.tiff]
