# Supplementary material for: Extraneural infection route restricts prion conformational variability and attenuates the impact of quaternary structure on infectivity
Source: PLoS Pathog. 2024 Jul 8;20(7):e1012370. doi: 10.1371/journal.ppat.1012370 (PMC11257401; doi:10.1371/journal.ppat.1012370)
Supplement: Attachment — Submitted filename: Chang et al Response Plos Pathogens R2 21062024.pdf [file ppat.1012370.s009.pdf]

Response to reviews

We are grateful to the reviewers for reading and evaluating our manuscript, and we were relieved to see that Reviewer 3 was satisfied by our explanations of experimental procedures. Please see below in detail further changes we have made to our manuscript to address remaining concerns.

Reviewer #1:

The responses from the authors to the concerns regarding the data in figure 2 raise concerns regarding rigor. For the vaculation profile a n of two animals is insufficient to make meaningful conclusions. It is unclear how a SEM can be represented in the figure from an n of two. The differences measured could simply reflect animal variation and conclusions based on this are suspect.

The main conclusion from former Figure 2 was that i.p. inoculated mice, independent of inoculum, have lower PrP^{Sc} deposit and vacuolation scores in most brain regions except for medulla, cerebellum, thalamus. We do recognize the concerns raised about the small number of mice analysed. Therefore, we decided to solidify the aforementioned conclusion by adding new Figure 2, for which we combined PrP^{Sc} deposit and vacuolation scores obtained from all i.p. and i.c. inoculated animals analysed (n = 4), and restricted our conclusion to differences between i.p. and i.c. inoculation irrespective of strain. We moved former Figure 2 into the supplement as new S2 Figure, and we considerably softened our language when referring to S2 Figure in lines 130 – 150, even pointing out the limitation of the small number of mice not allowing conclusions about strain-specific differences:

*Lines 133 – 138: "More variation between the two inocula was observed in i.c. than i.p. infected mice for both PrP^{Sc} deposits and vacuolation; however, the small number of mice analysed from each individual group does not allow us to draw conclusions about strain-specific differences. Therefore, we focused our analysis on comparing PrP^{Sc} deposits and vacuolation profiles between the two inoculation routes irrespective of inoculum (**Figure 2**).*

These changes do not alter the previous conclusion, but hopefully will convince this referee of the rigor of our analysis.

The SEM is derived from individual blinded readings (e.g. in new Figure 2, n = 4 individual mice, 3 reading scores for an n = 12 in the graph). We apologise for the lack of clarity, these details have been added to the methodology.

Second, for the PrPSc deposit data, the authors indicate in the rebuttal that "Between 2 and 4 sections were read 3 times from each mouse brain and averaged." The number of reads is fine, but examining 2-4 sections per animal is inadequate. Subtle differences in the anatomical location between the sections and differences in IHC performance could explain differences rather than a true difference in PrPSc distribution. Overall, conclusions based on figure 2 in its current form are not completely supported.

Possibly our previous description of the methodology was not clear enough, and we have now added information the consecutive brain sections were used. While in most of the literature the methodology is not described in much detail, our method aligns with published methods by e.g. Duque-Velasquez J Virol 2015; Fraser and Dickinson, 1968.
