# Supplementary material for: Extraneural infection route restricts prion conformational variability and attenuates the impact of quaternary structure on infectivity
Source: PLoS Pathog. 2024 Jul 8;20(7):e1012370. doi: 10.1371/journal.ppat.1012370 (PMC11257401; doi:10.1371/journal.ppat.1012370)
Supplement: Attachment — Submitted filename: Review for Plos Pathogens.docx [file ppat.1012370.s007.docx]

The authors demonstrated that the two distinct chronic wasting disease strains were similar when injected intracerebrally but differ within in the brain and spinal cord when injected intraperitoneally further proving CWD extra neuronal propagation is unique. These findings are very interesting and show that the more natural mode of transmission, peripheral exposure, is highly stable conformationally within the brain. Many of these findings are based on immunohistochemistry and morphological changes however this analysis was performed on a small sample size and would need to be increased in order to make these claims.

However, the manuscript can be improved and more convincing if the authors address the following issues:

1.) Figures 2 and 5 are showing pathological scoring data, for both PrP^Sc^ deposits and vacuolation, of different brain regions in brain samples from a minimum of 2 mice for each group. The number of brains analyzed should increase for rigor of the analysis to at least an N of 3 or 4 to properly interpret this data. Currently this is too small of a sample size to make any conclusions.
2.) Is the deposition of PrP^Sc^ found equally on both hemispheres of the brain or is one hemisphere more affected than the other? Does this differ between ip and ic inoculations. This information can also add information about these two different isolates and the possible different strains ic and ip are producing.
3.) The fractionated PrP^Sc^ was shown to decrease variance in survival with ip indicating that peripheral infection is independent of quaternary structure. This is a significant and interesting finding. Would other peripheral organs (ie. spleen or lymph) have a similar PrP^Sc^ immunoblot and conformational stability profile as the brain and spinal cord in the ip inoculated animals? This would help to explain if this is a nervous tissue or periphery vs central finding. This data would be beneficial to understand more about the tissue specific factors involved.
4.) It would be beneficial to discuss the implications and possible mechanistic/ pathological reasons the Wisc-1 isolate survival time did not correlated to amount of PrP^Sc^. This is an important difference found in these two relatively similar isolates and should be discussed further.
5.) It would be important to test this extaneuronal phenomenon in other isolates of CWD. This should be discussed or mentioned as a limitation as these findings are in two of many natural CWD isolates.
