# Supplementary material for: Extraneural infection route restricts prion conformational variability and attenuates the impact of quaternary structure on infectivity
Source: PLoS Pathog. 2024 Jul 8;20(7):e1012370. doi: 10.1371/journal.ppat.1012370 (PMC11257401; doi:10.1371/journal.ppat.1012370)
Supplement: Attachment — Submitted filename: Chang et al Response PPATHOGENS-D-24-00641 R1 16052024.docx [file ppat.1012370.s008.docx]

**Response to reviews**

First of all, we would like to thank the reviewers for their time to evaluate our manuscript. We are grateful for all the positive comments and the constructive suggestions, which have helped us to strongly improve the clarity of our manuscript. Please fine below a point-by-point response to all concerns. Please note that line numbers refer to the ‘tracked changes’ version of the manuscript.

**Part II – Major Issues**

**Reviewer #1:** (No Response)

**Reviewer #2:** (No Response)

**Reviewer #3**:

1.) Figures 2 and 5 are showing pathological scoring data, for both PrPSc deposits and vacuolation, of different brain regions in brain samples from a minimum of 2 mice for each group. The number of brains analyzed should increase for rigor of the analysis to at least an N of 3 or 4 to properly interpret this data. Currently this is too small of a sample size to make any conclusions.

Response: Unfortunately, we did not reserve more brain samples for histological analysis to save as many brains as possible for biochemical analysis. Overall, analysis was performed as previously described (refs 58 – 60). While brains of only two individual mice were analysed, these constitute 25 – 40 % of the number of mice/group that reached a terminal disease endpoint. To generate meaningful data, multiple sections from each brain were analysed and averaged, along with controls to ensure specificity. Since scoring is semi-quantitative, we omitted statistical analysis to avoid the impression of quantitative data.

2.) The fractionated PrPSc was shown to decrease variance in survival with ip indicating that peripheral infection is independent of quaternary structure. This is a significant and interesting finding. Would other peripheral organs (ie. spleen or lymph) have a similar PrPSc immunoblot and conformational stability profile as the brain and spinal cord in the ip inoculated animals? This would help to explain if this is a nervous tissue or periphery vs central finding. This data would be beneficial to understand more about the tissue specific factors involved.

Response: This is a great suggestion. Notably, we have not analysed conformational stability from any of the tissues (brain, spinal cord or spleen) of the mice inoculated with fractionated PrP^Sc^, and feel that this additional analyses would be beyond of the scope of the current manuscript.

**Part III – Minor Issues**
**Reviewer #1:**

In general, the authors are encouraged to provide p values where significance is indicated and to provide information on the number of animals examined for each experimental group.

Response: We apologize for the lack of clarity and have now added the number of animals analysed along with the p-values in the figure legends.

The observed differences in incubation time, PrPSc abundance and neuropathology between i.c. and i.p. infected animals could be due to route of infection and not necessarily a change in strain properties. However, the PrPSc conformational stability data argues against this explanation and is very intriguing. In the manuscript the authors present this data in figure 3. It is unclear from the material and methods or the results section how many animals per each group were tested or how many technical replicates were performed. Additionally, the GdnHCl half values and SEM are not reported or are statistical significance p values reported. Inclusion of these would strengthen the conclusions of the authors. Additionally, in figure 3, panels E and F, the 0M values are lower in spinal cord vs. brain. Do the authors think differences in the starting amount of PrPSc is contributing to the differences observed?

Response: For the conformational stability analysis in Figure 3, basically every animal included in Figure 1A has been analysed (n = 6 mice for i.c. inoculation, n = 8 for i.p. inoculation). Analysis was performed in triplicate, and two technical replicates were done. The lower starting values of spinal cord vs. brain at 0 M is anomaly of the curve fitting software, the actual data points are still at a ratio of 1 for both tissue types. This phenomenon is commonly observed and published as such also by others, e.g reference 47, Bian et al., PNAS 2019.

Regarding the spinal cord, additional details on the collection of the spinal cord are important for a complete understanding of lines 276-286. What level of the spinal cord was collected and did this vary between animals? In the discussion, the authors suggest that strain adaptation is occurring in the spinal cord prior to transport to the brain. Based on the anatomy of neuroinvasion following i.p. inoculation this would be expected to occur in the IML of the thoracic spinal cord, replication in these cells followed by retrograde axonal transport to the brain. Clarification of the spinal cord collection will mitigate this concern. Also, at terminal disease the cord from i.p. infected animals will contain a mixture of IML originating prions and those that migrate from the brain, the authors should consider this in the interpretation as the effect in the IML may be greater than observed in the cord homogenate.

Response: We collected the entire spinal cord, but we agree that dissecting it at different levels would be very informative; however, to be able to perform conformational stability analysis, a greater amount of homogenate is needed than what we would be able to get from individual sections of mouse spinal cords, therefore, we opted to analysis whole spinal cord homogenates. Further clarification and discussion of the excellent point about mixture of anterogradely and retrogradely transported prions has been added in lines 272 – 276.

Two additional pieces of data, if available, would further strengthen the manuscript. The idea of prion adaptation during the pathogenesis of disease is very interesting and important, do the authors have any insight into what is occurring in the spleen? Second, have the authors taken the i.p. passaged material and performed a serial i.c. passage to see if the strain properties are maintained or if it reverts back to the original strain phenotype?

Response: We agree that both points raised would be interesting to investigate; we did not analyse spleen tissues as variation between spleen and obex tissues have been already reported by Wagner et al, 2022 (ref 51). We did not perform second passage and have added this as a potential limitation of our study in lines 325 – 330.

**Reviewer #2:**

1. It would be helpful if the source of unfractionated inoculum for the initial transmission experiments could be stated – in particular, the genotype of the WTD infected with either Wisc1 or 116AG.

Response: This information has been added in the Materials and Methods section, in lines 342 – 344, along with references that describe the characterisation of these isolates in detail (refs 26 – 29).

2. Although Wisc1 and 116AG are described as being distinct strains of CWD, their incubation periods and distribution of PrPSc/vacuolation in the brain are quite similar in Prnp.Cer.Wt mice, whether injected i.c. or i.p. The major strain-specific differences between mice inoculated i.c. seem to be in the vacuolation profile (Fig 2D), and in PrPSc conformational stability/relative PK resistance. Can the authors comment on whether these differences reflect the strain-specific characteristics observed when these specific CWD strains are passaged in mouse lines over-expressing CerPrP?

Response: More detail comparing the differences to those isolates passaged in mouse lines overexpressing CerPrP has been added in lines 229 – 231, along with references (28, 29); in such a model, we also observed differences in PK resistance and conformational stability, an in addition, in survival times. In these former studies, we did not perform formal scoring of PrP^Sc^ distribution and vacuolation.

3. The scoring system for PrPSc deposition in different brain regions is not described in detail in the methods, nor citation provided for a published method. The methods state that the images were analysed using NDP.view 2 software – how? By eye? If so, how are the different scores specifically defined (does it take account of different types of staining e.g. plaques, intraneuronal, glial, ependymal etc), and is a known reference slide included in each IHC run as a comparator to account for inter-run variability in staining? Each analysed sample was scored blindly three times – was all scoring done by one individual, or was more than one person responsible (similar questions for vacuolation scoring)?

Response: We apologize for the lack of clarity and have now added more detail in the Material and Methods section (lines 407 – 425), along with references (58-60). Reading was done on scanned images mostly by one reader with additional blinded reading of select slides by a second reader. Between 2 and 4 sections were read 3 times from each mouse brain and averaged. The IHC scores reflect intensity of staining. Control slides were included from the same mouse line.

4. Figure 2 – it would be helpful if the legend could state the numbers of animals analysed to provide the scores. Was any statistical analysis performed to determine the significance of observed differences? Error bars are stated as SEM, but in the methods it says that scores are reported as mean ± standard deviation – please clarify.

Response: The number of mice has been added, and the methods section has been corrected to state that error bars represent SEM. No statistical analysis was performed as the scoring is semi-quantitative, and we only analysed the brains of two mice/group.

5. Conformational stability of PrPSc (Figure 3) - Values are expressed as a ratio of baseline signal at 0M Gdn-HCl – this suggests that all curves should start at a value of 1, and the fact that some do not makes it more difficult to compare curves in some cases. Can the authors explain why this is? Also, although an indication of statistical methods and key to p values is provided in the legend to Fig 3, there are no symbols (asterisks) within the figure charts to indicate where statistically significant differences were found.

Response: We apologize for the lack of clarity, all information has been added to the figure and figure legends.

6. The results with the fractionated brain homogenates are somewhat puzzling. The most likely explanation for the variation in incubation periods observed with ic inoculation of fractions would be differences in titre, and to some extent this seems to fit expectation, with the shortest IPs for fractions with relatively small aggregates, and longer IPs for fractions containing larger aggregates. If this is the case, a similar pattern might be expected in the mice inoculated ip with the same fractions, but this is not the case. Could this be a dose effect? In that ip inoculated mice receive a much larger dose (volume)? Or does it suggest that favourable conditions for peripheral selection of a neuroinvasive sub-strain of ip injected prions exist across a wide range of infectivity/PrPSc titres? Were end-point titrations of Wisc1 and 116AG by ic and ip injection performed in Prnp.Cer.Wt mice at any point, and if so, could this be helpful in relating incubation periods to titre for the fractionated brains?

Response: We agree that it might be helpful to determine titers of unfractionated inocula to obtain more information, however, we did not perform end-point titrations. Our main argument why we do not need to determine absolute infectious titers is that we only compare profiles of survival, and that variations with respect to relative amount of PrP^Sc^ are the same in i.c. and i.p. inoculation (see lines 301 – 306). We hope this addressed the concern.

7. Figure 5 – IHC profiles from mice inoculated with different brain fractions. The authors point out that for Wisc1, the profiles are fairly consistent across fractions in mice inoculated by both routes, whereas there is greater variability for 116AG, particularly for the bottom fraction. Can they comment on whether they consider this to be due to isolation of different strains or sub-strains in the different fractions of 116AG? Do lesion profiles from scoring vacuolation give similar results? Again, would be helpful to have the numbers of mice represented in the plots indicated in the figure legend (n=?).

Response: We have added the number of mice analysed in the figure legend. Here, we did not perform vacuolation analysis. In terms of substrains of 116AG in different fractions, we have published previously that 116AG consists of two substrains (ref 29), and have used here 4^th^ passage of an uncloned version of one of the substrains; however, keeping in mind the study published by Gunnels et al, 2023 (ref 49) that even in cloned strains there is evidence for substrain diversity, a substrain portion is a possibility here. We have added a sentence to the discussion in lines 309 - 311.

8. In the discussion (lines 306-309), the authors suggest: “Spleen tissue expresses about 20-fold lower levels of PrPC than the brain (50). This possibly results in better accessibility and enhanced interaction of PrPC with a wide range of PrPSc aggregates, attenuating variability between PrPSc aggregate replication efficiency.” It is hard to follow the reasoning – would a lower concentration of PrPC not suggest a reduced number of sites for PrPSc/PrPC interactions? This could be clarified. Is it possible that competition between (sub)-strains within the brains of ic injected mice, leads to greater variability in outcomes, whereas the neuroinvasive sub-strain selected during ip infection can replicate more consistently and reproducibly when it reaches the brain?

Response: We have added more clarification to the line of thoughts on the difference in PrPC expression levels between spleen and brain in lines 315 – 316, arguing that PrPC expressed at higher levels might cluster and lead to steric hindrance of PrPC-PrPSc interations (lines 320 -322). We have included a discussion sentence about the possibility of (sub)strain competition in i.c. inoculated animals in lines 252 – 253.

**Reviewer #3:**

1.) Is the deposition of PrPSc found equally on both hemispheres of the brain or is one hemisphere more affected than the other? Does this differ between ip and ic inoculations. This information can also add information about these two different isolates and the possible different strains ic and ip are producing.

Response: We usually use half brains dissected sagittal for formalin fixation and IHC, but have never observed unilateral distribution (i.e. negative brain hemispheres). Therefore, unfortunately we cannot comment about differences in intensity between both hemispheres.

2.) It would be beneficial to discuss the implications and possible mechanistic/ pathological reasons the Wisc-1 isolate survival time did not correlated to amount of PrPSc. This is an important difference found in these two relatively similar isolates and should be discussed further.

Response: We have added a comment in lines 118 – 120, clarifying that our analysis here only focused on PK-resistant PrP^Sc^; however, this does not correlate to the total amount of PrP^Sc^, which would have to take PK-sensitive PrP^Sc^ into account as well. Since we did not analyse total PrP^Sc^, it is challenging to draw conclusions added a comment wrt PrPres versus PrP^Sc^ including PrPsen in lines 118 – 119.

3.) It would be important to test this extaneuronal phenomenon in other isolates of CWD. This should be discussed or mentioned as a limitation as these findings are in two of many natural CWD isolates.

Response: We agree and have added this in a new paragraph in the discussion describing potential limitations of our study in lines 324 – 328.
